# Diseases of the Nervous System

**Published:** 1905-05-20

**Authors:** 


### DISEASES OF THE NERVOUS SYSTEM.

(*Continued from page* 91.)

**Kernig's sign** was by its discoverer believed to be symptomatic of cerebro-spinal meningitis. More extended observations, however, have shown that it is present in many other diseases, and a summary of our present knowledge has recently been published by Sainton and Voisin.[3] The original method of eliciting the sign was to make the patient sit on the edge of his bed with the feet hanging down; if the sign is positive one cannot straighten the leg at the knee owing to contracture of the flexors of the knee. The usually adopted method now is to make the patient lie on his side and then to flex the thigh on the pelvis the knee being kept extended; if the sign is positive this is impossible. The conditions in which the sign has been found are:—1. Acute cerebro-spinal meningitis and tuberculous meningitis, in which it is present in more than three-fourths of the cases. 2. Meningeal hæmorrhage. To distinguish between inflammatory and hæmorrhagic conditions it is necessary to practise lumbar puncture, and examine the cerebro-spinal fluid. 3. Infectious diseases, especially typhoid fever, and also in pneumonia, where there are no other indications of meningitis. 4. Certain forms of intoxications, notably uræmia. 5. Diseases of the nervous system, apart from those of infectious origin—*e.g.* a few cases of cerebral hæmorrhage, certain cord affections, sciatica. There is a general concensus of opinion that the sign is due to contracture of the flexors of the knee, a hypertonus of these muscles, and that it is simply an exaggeration of a normal phenomenon. This hypertonus has been explained in various ways—1. As due to increased tension of the cerebro-spinal fluid; this, however, though present in some cases is certainly in many quite absent. 2. Due to an irritative lesion of the cord or its roots. But in many cases no alteration of the cauda equina, spinal cord, meninges, or cerebro-spinal fluid has been found. 3. A reflex due to pain. This view presupposes the existence of a local irritative lesion of the sacral plexus, which has not been found in all cases. No one of these views is wide enough to include all cases, and the sign is probably a reflex phenomenon due to various causes acting on the so-called excito-reflex cells of the cord. These influences may come from the periphery (as in sciatica), from the brain (as in hemiplegia), or from local inflammation of the meninges, or from various forms of intoxication acting on the cord without necessarily causing true meningitis.

**Amaurotic Family Idiocy.**—A complete report of a case of this rare disease has been published by McKee, Buchanan, Shumway, and Spiller.[4] The child, aged one year, was first brought to the hospital suffering from rickets. There was no history of insanity, imbecility, tuberculosis, or syphilis, or amaurotic idiocy in the family of either parent. Six months later the child became blind, ceased to take notice of his surroundings, very somnolent. There was paresis of the neck muscles, and a spastic condition of the limbs, with exaggeration of the tendon-jerks and extensor plantar reflexes. The left pupil was widely dilated and did not respond to light, the right was moderately dilated and responded. The ocular fundi were characteristic of the condition. The media were clear, the optic discs atrophic and bluish-white in colour, the vessels very much reduced in size, especially the arteries. The fovea showed as a dark-red oval with sharply-defined edges, the colour corresponding to that of the veins. Surrounding the fovea was a large greenish-white oval patch, and gradually fading away towards the periphery. The diameter of this white patch was about one and a half discs' diameter. The child died in February 1904 from catarrhal pneumonia. A 10 per cent. solution of formalin was injected into the posterior chamber of each eye a little time after death, and the eyes and central nervous system subsequently removed and examined. It was found that the ganglion cells of the retina were in an advanced state of degeneration. Many had disappeared entirely, so that in the macula, where the cells are ordinarily present in rows of from three to six, only a single interrupted row could be seen. In those that remained the cell outlines were irregular and without any dendritic processes, the Nissl bodies had disappeared, and the nucleus was distorted. The retina as a whole was thinner than normal, and the thinning seemed to be at the expense of each of the layers. The sections of the optic nerve revealed an entire absence of normal fibres in its temporal half, and a great reduction in those on its nasal side. Sections of the chiasma and optic tract showed complete atrophy of all the nerve fibres. The essential changes in the eyes are thus degeneration of the ganglion cells of the retina and of the nerve-fibres of the optic tracts and nerves, which are genetically a portion of the central nervous system. Examination of the central nervous system showed that the chromophilic elements of the nerve-cells had disappeared, so that not a single normal cell was found. The Marchi method showed the most intense alteration of the white matter of the brain and cord. The changes in the central nervous system and retinæ were thus similar—a degeneration of the nerve-cells with resulting degeneration of the nerve-fibres. The causes of this are obscure —one view is that they are hereditary, that the disease is an arrest of development, the other that the process is the result of some intoxication. In favour of the first view is the fact that the disease is almost always in Jewish families, and in some cases more than one member has been affected.

[3] Gaz. des Hôpitaux, Aug. 27, 1904. [4] Amer. Jour. Med. Sc., Jan. 1905.

